# Transition to Quorum Sensing in an *Agrobacterium* Population: A Stochastic Model

**DOI:** 10.1371/journal.pcbi.0010037

**Published:** 2005-09-16

**Authors:** Andrew B Goryachev, Da-Jun Toh, Keng Boon Wee, Travis Lee, Hai-Bao Zhang, Lian-Hui Zhang

**Affiliations:** 1 Systems Biology Group, Bioinformatics Institute, Singapore; 2 Institute of Molecular and Cellular Biology, Singapore; Hebrew University of Jerusalem, Israel

## Abstract

Understanding of the intracellular molecular machinery that is responsible for the complex collective behavior of multicellular populations is an exigent problem of modern biology. Quorum sensing, which allows bacteria to activate genetic programs cooperatively, provides an instructive and tractable example illuminating the causal relationships between the molecular organization of gene networks and the complex phenotypes they control. In this work we—to our knowledge for the first time—present a detailed model of the population-wide transition to quorum sensing using the example of *Agrobacterium tumefaciens*. We construct a model describing the Ti plasmid quorum-sensing gene network and demonstrate that it behaves as an “on–off” gene expression switch that is robust to molecular noise and that activates the plasmid conjugation program in response to the increase in autoinducer concentration. This intracellular model is then incorporated into an agent-based stochastic population model that also describes bacterial motion, cell division, and chemical communication. Simulating the transition to quorum sensing in a liquid medium and biofilm, we explain the experimentally observed gradual manifestation of the quorum-sensing phenotype by showing that the transition of individual model cells into the “on” state is spread stochastically over a broad range of autoinducer concentrations. At the same time, the population-averaged values of critical autoinducer concentration and the threshold population density are shown to be robust to variability between individual cells, predictable and specific to particular growth conditions. Our modeling approach connects intracellular and population scales of the quorum-sensing phenomenon and provides plausible answers to the long-standing questions regarding the ecological and evolutionary significance of the phenomenon. Thus, we demonstrate that the transition to quorum sensing requires a much higher threshold cell density in liquid medium than in biofilm, and on this basis we hypothesize that in *Agrobacterium* quorum sensing serves as the detector of biofilm formation.

## Synopsis

Understanding the interplay between the extracellular environment and intracellular decision circuitry of a cell is important but is an arduous goal to achieve since many interacting factors, difficult to measure and control in experiment, are involved. The authors address this problem by means of computational modeling using the example of a bacterial population that cooperatively switches on a common gene expression program if a certain critical population density is achieved. They developed a detailed model of the intracellular control network and demonstrated that it can operate as an “on–off” gene expression switch that is sensitive to environmental control and yet highly robust to intracellular molecular noise. The population-wide transition is further modeled using a novel method in which each bacterium is given a unique copy of an intracellular network. This approach, which allows monitoring of both the dynamics of individual cells and population behavior, provides an explanation for the gradual appearance of the transition to the “on” state that has been observed in experiments, and quantitatively predicts the critical value of the population density at which this transition occurs. Unexpectedly, a comparison of the cell densities required for the transition in different environmental conditions brought about a hypothesis regarding the previously elusive ecological and evolutionary function of this cooperative phenomenon.

## Introduction

Molecular networks, which integrate signal transduction and gene expression into the unified decision circuitry, are ultimately responsible for the realization of all life activities of biological cells including internal developmental programs and responses to environmental factors. One of the main challenges of systems biology is to uncover and understand the relationships between the properties of these molecular circuits and the macroscopic cellular phenotypes that are controlled by them [1]. Particularly important are the phenotypes involving interaction and cooperative action of multiple cells. The mapping of networks onto phenotypes is still difficult to accomplish in multicellular eukaryotic organisms owing to their staggering complexity. Less complex and more experimentally accessible prokaryotic organisms became the systems of choice for “dissecting social behavior at the genetic level” [2]. The phenomenon of bacterial quorum sensing (QS) gives us a particularly unique opportunity to follow the causal relationships from molecular circuitry to cooperative population dynamics.

QS refers to the ability of bacterial populations to collectively activate certain gene expression programs, e.g., toxin release or antibiotic production, once some critical population density has been reached. QS is found in a vast variety of bacterial species and has been extensively studied experimentally [3–6]. In Gram-negative bacteria, the QS phenomenon is usually controlled by a small gene expression network that functions as an environmentally activated “on–off” gene expression switch [5,6] whose operation is analogous to that of radar. At the low cell density that normally corresponds to the “off” switch state, a key transcription factor required for the expression of proteins responsible for the phenotype is suppressed. At the same time, the cell steadily produces a small amount of the QS signaling molecule, termed the autoinducer, that can freely diffuse in and out of the cell. While the population density is low, most of the autoinducer molecules are washed out and dispersed in the environment by diffusion. As the cell density grows, more molecules of autoinducer enter the bacterium from outside. Once certain cell “quorum” is reached, the inbound autoinducer signal triggers the transition of the QS network to the “on” state, resulting in the production of the transcription factor and the expression of the target genes.

This transition on both intracellular and population-wide scales is the focus of our study. We investigate the phenomenon of QS in the soil-dwelling plant pathogen *Agrobacterium tumefaciens,* the causative agent of crown gall disease [7]. Bacteria of this species often harbor Ti (tumor-inducing) plasmids that endow their hosts with the unique ability to genetically modify susceptible plants through a cross-kingdom DNA transfer. Like many other soil bacteria, *Agrobacterium* is chemotactic to exudates released by plant wounds and is capable of catabolizing various nutrients that leave injured plant roots. Once bacteria form physical contact with the surface of the wound, Ti plasmids offer their hosts an extraordinary advantage over their plasmidless competitors. A fragment of the plasmid, termed the *vir* region, is injected into the plant cell in the form of a virion-like complex and is stably incorporated into the plant genome [7]. One of the imported genes is responsible for the synthesis of opines, a class of low-molecular-weight nitrogen-rich metabolites, that can be utilized as a nutrient only by the bacteria that harbor the Ti plasmid. Other transferred genes cause a vigorous proliferation of infected plant cells that eventually results in the formation of a characteristic gall tumor. Once productive infection is established, Ti plasmids attempt to propagate themselves into the plasmidless bacteria of the same or related species by means of genetic conjugation. It has been shown that the conjugal transfer of Ti plasmids requires the QS phenomenon [8].

Functional significance of QS for the control of Ti plasmid conjugation remains an ecological and evolutionary puzzle. It is widely believed [5,6] that QS controls processes, such as production of toxins and antibiotics, that are either inefficient or devoid of adaptive value if not performed on a population scale. Thus, the fact that the establishment of QS is upstream of the initiation of conjugation seems to imply that plasmids await the critical density of donors to collectively begin transfer to recipients. Since multiple donors cannot cooperate in DNA transfer, the necessity for collective action does not seem to be relevant in our case. Instead, to increase the probability of successful conjugation it would appear beneficial to exceed a certain number of recipients per donor. However the density of plasmidless recipients cannot be estimated using QS since they do not produce the autoinducer. This seemingly paradoxical situation may imply that our understanding of the biological function of QS is not yet complete. Indeed, an alternative function for QS as a sensor of the volume enclosing the bacteria has also been proposed [9]. To answer what bacteria really measure using QS in each particular situation, it is necessary to consider the ecologically relevant conditions of bacterial growth [2].

An experimental approach to this problem is often complicated by the technical difficulty of work in real ecosystems. On the other hand, mathematical modeling can significantly aid and complement experimental methods in answering biological questions that involve spatial and temporal scales of the QS phenomenon. Some aspects of either intracellular [10–13] or population [14–16] dynamics have been mathematically modeled to gain insight into the QS phenomenon in *Pseudomonas aeruginosa* and *Vibrio fischeri*. However, because of the lack of detailed molecular information, experimentally testable conclusions on the connections between intracellular and population dynamics have rarely been made. Here we develop a multi-level modeling approach that describes both the intracellular and the population-wide dynamics and allows us to follow the connections between them explicitly. Although much has been learned about the molecular details of the *Agrobacterium* QS network, it is not always clear what functions they perform. Here we construct a detailed model of the QS network in *Agrobacterium* and analyze it both quantitatively and qualitatively. We demonstrate that the network possesses properties of the on–off gene expression switch robust to molecular noise. We further develop a population-scale model that incorporates bacterial motion, cell division, and chemical communication while explicitly considering the individual intracellular dynamics of each cell. This allows us to describe the transition to QS on both cellular and population scales and quantitatively predict the values of critical autoinducer concentration and threshold cell density as functions of various intracellular and environmental parameters. Finally, comparing feasibility of the transition to QS in homogeneous medium and biofilm, we present a hypothesis explaining the ecological and evolutionary roles of QS in regulation of Ti plasmid conjugal transfer.

### The QS Network and Model Assumptions

**Figure 1:**
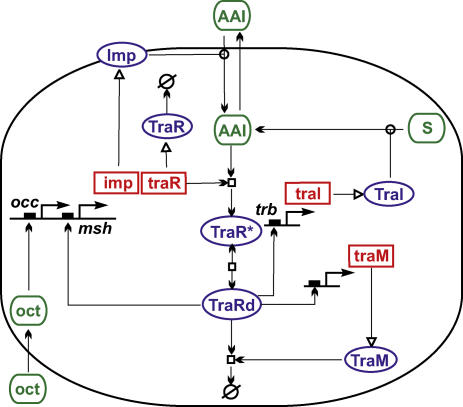
Quorum-Sensing Network of an Octopine-Type *Agrobacterium* Ti Plasmid Blue ellipses represent proteins, red rectangles indicate mRNA species, and green flattened circles denote metabolites. Open circle arrowheads represent enzymatic activation of a reaction or transport, and open triangle arrowheads denote translation. Essential bimolecular reactions are shown explicitly as open squares. S represents two substrates of the autoinducer synthetase TraI, and Ø denotes protein degradation.

All genes that are thought to constitute the QS network are located on the Ti plasmid itself [7]. The entire QS network is controlled upstream by the availability of the plant-produced opines to ensure that energetically expensive conjugation machinery is activated only after the establishment of a successful plant wound infection. Based on the chemical nature of the encoded opines, Ti plasmids are divided into two major types [7], of which we consider only the octopine type. We reconstructed the layout of the QS network for the octopine-type Ti plasmids from the published experimental data (Figure 1). In this plasmid class, octopine molecules that are imported through the cell wall eventually cause activation of transcription from the operon *occ* [17]. In the model, we assume that octopine is constitutively available at the saturating concentration that results in the maximal rate of *occ* transcription. The last open reading frame of this operon codes for the QS transcription activator TraR. Binding of TraR to its cognate autoinducer is thought to occur only within a narrow window of time during *traR* mRNA translation when the newly formed protein chain tightly winds around a single molecule of *Agrobacterium* autoinducer (AAI) [18–20]. This total engulfment of AAI molecule makes formation of the TraR–AAI complex (TraR*) practically irreversible. Furthermore, TraR protein translated in the absence of AAI is misfolded, insoluble, and unable to bind AAI [18,20]. This has an important consequence in that the rate of production of TraR* depends on the concentrations of *traR* mRNA and AAI and does not depend on the accumulation of misfolded TraR protein, as explicitly shown in Figure 1. Once formed, TraR* quickly dimerizes to form a stable transcriptionally active TraR* dimer (TraRd) with a relatively short half-life of 35 min [18]. TraRd is capable of activating a number of operons that encode proteins necessary for conjugation. The first open reading frame of the *trb* operon codes for the acyl-homoserine lactone synthetase TraI, which utilizes two metabolites abundant in the bacterial cell to create AAI [21]. Since our model considers transition to QS in the mostly nutrient-rich, stress-free conditions of an optimized growth medium, we assume that the substrates of TraI are present in excess and their concentrations do not limit the rate of AAI production. Both *traR* and *traI* were shown to be expressed at some low constitutive rate even in the absence of octopine [7]. The TraR–TraI couple constitutes the classic QS positive feedback loop found in many Gram-negative bacteria. Additional feedback loops that also involve other components of the QS network are specific for *Agrobacterium*. Thus, negative control of QS is provided by the antiactivator *traM,* whose transcription is directly activated by TraRd [22]. TraM effectively sequesters TraRd through the formation of a very stable complex in which TraRd is unable to bind DNA [23,24]. Recently, a number of authors reported that, like TraR, TraM also forms a dimer [25–27]. The stoichiometry of the reaction between TraR and TraM, however, remains controversial [25–27]. In our model we follow the original hypothesis of Swiderska et al. [24], which assumes that the complex consists of one TraRd and one monomer of TraM. This hypothesis is partially supported by Chen et al. [26], who showed that the TraM dimer must dissociate to form a complex with TraR. Under these assumptions we disregard dimerization of TraM as not affecting the network behavior. An additional positive feedback loop arises because TraRd activates transcription of the *msh* operon, which is a suboperon of *occ* that contains *traR* itself.

Several lines of evidence suggest that active transporters facilitate traffic of the QS signaling molecules through the cell wall in a number of bacterial species including *Agrobacterium* [28–30]. In our model, we explore the hypothesis that AAI is imported from the environment by an active pump that is also under the transcriptional control of TraRd. Indeed, the *msh* operon contains five open reading frames (*ophABCDE*) that encode a putative ABC-type importer whose function is not completely understood but that has been hypothesized to be an active transporter of AAI into the cell [30]. Taking into consideration this uncertainty, the putative AAI importer in the model is denoted simply as Imp.

## Results/Discussion

### The QS Network Can Operate as a Gene Expression Switch

**Figure 2:**
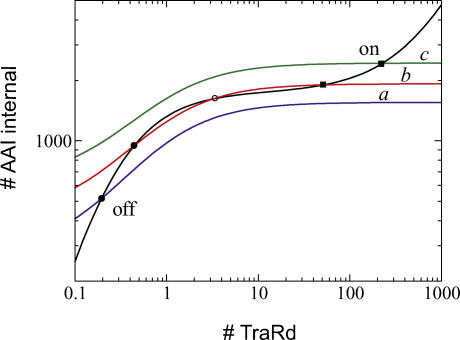
Nullclines of the Reduced Quorum-Sensing Model at Several Extracellular Concentrations of AAI Nullclines represent lines on which the respective variable does not change with time (e.g., *d*[*traRd*]/*dt* = 0 on the TraRd nullcline), and their intersections correspond to the stationary states of the model. The TraRd nullcline is shown as a solid black line and the *A_i_* nullcline at three values of *A_e_*—(a) 35 nM, (b) 67 nM, and (c) 142 nM—is shown in color. Positions of stable stationary states are marked by filled circles for the off network state and by filled boxes for the on state. The open circle indicates the unstable steady state of a saddle type.

We first asked whether the intracellular model constructed by us purely from the individual molecular interactions indeed describes known biological properties of the QS network. Although numerical simulation of the full model can answer this question in principle, it does not bring qualitative insight into the system's behavior. Thus, we reduced the full model to only two nonlinear differential equations describing TraRd and intracellular AAI that are readily amenable to qualitative analysis (see Materials and Methods for details). Figure 2 demonstrates that the QS network indeed possesses the property of an environmentally activated gene expression switch. Depending on the value of *A_e_,* the model possesses one or two stable stationary states. The only stationary state at a low extracellular concentration of AAI is characterized by a vanishing number of TraRd molecules. In fact, a copy number of TraRd significantly below one indicates that most of the time transcription factor is not present in the cell at all and the QS network is in the off state. As AAI accumulates in the environment, the position of the *A_i_* nullcline elevates so that first the on state is born at some value of *A_e_* and then the off state disappears at the critical extracellular concentration, thus triggering the transition to the on state. As a result of this transition, the copy number of TraRd dramatically rises from less than one to several hundreds, and the production of AAI increases nearly10-fold.

**Figure 3:**
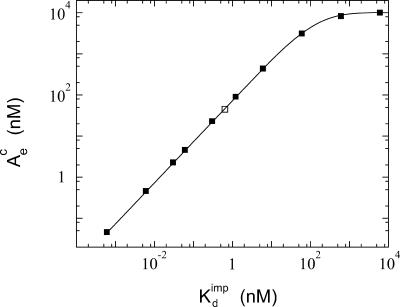
Dependence of the Critical Extracellular Concentration of Autoinducer 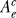 on the Autoinducer–Transporter Dissociation Coefficient 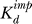 = *k_5_/k_4_* Predicted by the Intracellular Model The solid line is fitted to the computed data points indicated by the filled squares. The value of 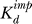
= 0.592 nM used throughout the simulations reported in this paper is shown as an empty square.

Sensitivity of the QS network to the changes in the extracellular concentration of AAI and consequently to the population density is defined by passive permeability of the cell wall to AAI and any active transport, if existent. Passive diffusion largely depends on the physical properties of the AAI molecule (e.g., length of the hydrocarbon chain) and cannot be controlled by the QS network. On the other hand, our model shows that an active importer, such as putative transporter Imp, can exert significant control over the transition threshold. Indeed, we found that the critical extracellular AAI concentration 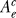 depends linearly on the AAI–Imp dissociation constant 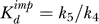 for a wide range of 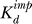 values (Figure 3). One may also speculate that the availability of such a pump could give the Ti plasmid evolutionary flexibility in the changing environment since a large multi-subunit protein complex can quickly accumulate mutations that potentially affect its transporter properties and consequently alter the transition threshold.

### TraM Is Necessary for the Existence of the Off State

Our model clearly demonstrates that the negative feedback provided by *traM* antiactivator is essential for the very existence of the off state in the QS network. Historically, *traM* was identified as a gene whose loss of function resulted in constitutive conjugation among the bacteria [22,31]. Indeed, the removal of the TraRd–TraM reaction from the model destroys the bistability of the QS network and permits only the on stationary state at any extracellular concentration of AAI. The model explains the mechanism of TraM action through the mutual exclusion between TraM and TraRd. In the off state, an appreciable number of TraM molecules (over 100 copies per cell) ensures that TraRd does not accumulate. During the transition to QS, rapidly created TraR* dimers sequester the TraM pool and reduce it to less than one molecule per cell in the on state. Production rate of the *traM* mRNA remains high in the on state, but the substantial surplus of TraRd guarantees that all newly synthesized TraM molecules are quickly sequestered. If this mechanism is inactivated by a mutation, the QS network amplifies any small number of TraRd complexes to the high on level even in the absence of exogenous AAI. This model prediction has been verified by genetic analysis. Strain K588, which produces AAI constitutively, was found to contain a point mutation in *traM* that renders the TraM protein inactive. Complementation of K588 with an intact *traM,* which was administered on a separate plasmid, restored the wild-type phenotype completely (L. H. Z., unpublished data).

### Sensitivity Analysis Reveals Critical Components of the QS Network

Additional information about the contribution of various components of the QS network to the control of the TraRd copy number can be gained from the analysis of sensitivity of the stationary concentration of TraRd to variation in the network parameters. One of the popular methods to assess sensitivity of molecular networks [32,33] is based on metabolic control analysis [34]. We computed the sensitivity of TraRd concentration to all network parameters in the off state at two values of *A_e_*, far from the transition to QS (*A_e_ =* 0) and in the bistable region (*A_e_* = 42 nM), as well as of the on state, deep into the region of its stability at *A_e_* = 120 nM. The three values of sensitivity coefficient calculated for each parameter as described in the Materials and Methods are given in Table S1. As expected, sensitivity to most parameters peaks in the on–off transition region. Processes responsible for the production and degradation of *traR* mRNA exert the most powerful control over the copy number of TraRd. However, the contribution of the positive feedback loop due to the transcription of the *msh* operon becomes significant in comparison with the input from the octopine-induced transcription of *occ* only during and after the transition to the on state. Interestingly, the sensitivity analysis shows that various components of the QS network contribute very differently into the control of TraRd in the on and off states. Thus, TraRd degradation is unimportant in the off state, when the copy number of the transcription activator is controlled by TraM, and acquires prominence in the on state, when sequestration by TraM becomes irrelevant. Likewise the Imp plays no role in the off state but exerts control over TraRd during the transition to QS. In contrast, the subsystem responsible for the production of TraI looses significance in the on state, when a large pool of this protein accumulates in the cell.

### The QS Network is Robust to Molecular Noise

**Figure 4:**
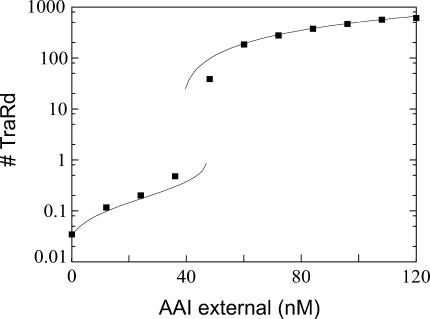
Transition to Quorum Sensing in the Stochastic Model of Intracellular Dynamics TraRd concentration averaged over 6 × 10^6^ s (filled squares) is plotted against extracellular concentration of AAI. The prediction of the deterministic model is shown as a solid line for comparison.

We next set out to investigate whether the sharp switch-like behavior of the QS network predicted by the deterministic model is preserved when fluctuations in the number of molecules are considered. To answer this question, we simulated the full intracellular model stochastically (see Materials and Methods) and compared the results with the deterministic solution. Figure 4 demonstrates that there is remarkably good agreement between the deterministic model and the behavior of the stochastic intracellular model averaged over a long observation time. The same results were obtained by averaging the behavior over many independent realizations.

Undetectable in the average value, fluctuations in the copy number of TraRd deserve special attention as they can dramatically affect the network behavior. In particular, the off state predicted by our model is biologically meaningful only if the fluctuations of TraRd are controlled as tightly as its average concentration. Rare but significant departures from the off state in the absence of the extracellular AAI signal (*A_e_* = 0) would result in spontaneous activation of genes encoding conjugation machinery under unfavorable conditions, bringing about selective penalty for the host bacteria. We performed a detailed analysis of the stability of the off state by investigating the TraRd probability density function at varying concentrations of extracellular AAI. The TraRd probability density function peaks at zero in the off state and decreases exponentially with the number of dimer molecules. At *A_e_* = 0, where the average TraRd concentration is 0.034, fluctuations of TraRd are practically negligible. Thus, two copies per cell are found with probability 0.001, while three copies are found with probability of only 6.6 × 10^−5^. This demonstrates that in the absence of external autoinducer the QS network maintains robust control of the fluctuations in the TraRd copy number and effectively prevents spontaneous transitions to the on state. At the same time, other molecular species whose copy number is not controlled by the QS network, e.g., TraI, TraM, and intracellular AAI, exhibit large-amplitude fluctuations around their average levels, in agreement with earlier reports for other molecular networks [35]. As *A_e_* increases, the fluctuations of TraRd also grow. At extracellular AAI concentrations in the range of 40–60 nM the network visits on and off states intermittently. For *A_e_* ≥ 70 nM, the model is found solely in the on state.

### Collective Robustness of the Population Transition to QS

**Figure 5:**
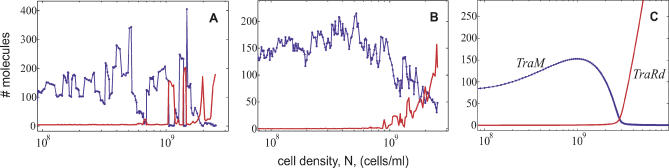
Transition of a Model Bacterial Population to Quorum Sensing in a Homogeneous Liquid Medium Intracellular concentrations of TraRd (thick red line) and TraM (thin blue line, filled circles) are plotted against the population density that exponentially grows with time. (A) Dynamics of an individual cell in the stochastic population model. (B) Dynamics of the stochastic population model averaged over ten bacteria. (C) Behavior of the deterministic population model.

We first investigated the transition to QS in the simplest case of a population growing exponentially in a homogeneous liquid medium. The stochastic population model was simulated to imitate actual population dynamics of motile bacteria in a small volume element (*V_e_* = 10^−5^ ml) of a liquid medium bulk. During approximately 7 h of simulation time, the population grew more than 100 times to reach the maximal density *N* = 2.52 × 10^9^ cells/ml. Figure 5 demonstrates the transition to QS in this system as detected by monitoring the intracellular dynamics of randomly selected bacterial cells. Observation of an individual bacterium over time shows that after the initial period of quiescent growth in the off state, TraRd exhibits sudden and fast switches to the on state (Figure 5A). These phases with variable duration and TraRd abundance alternate with the off phases in a random pattern until the cell finally settles in the on state. While the intracellular dynamics of individual bacteria appears to be totally erratic, the population average demonstrates an orderly, gradual transition to QS (Figure 5B). Moreover, ensemble-averaged behavior of the stochastic population model resembles that of the deterministic model (Figure 5C). For example, notice that in both cases prior to the transition to the on state TraM temporarily undergoes a maximum. To be precise, we can define the transition to QS to occur at the point where the lines for TraRd and TraM abundance intersect in Figure 5C. Then deterministic and stochastic models predict transition at the same critical value of extracellular autoinducer concentration, 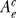 = 580 ± 3 nM. Thus, the collective transition to QS in the spatially homogeneous bacterial population, as defined by the ensemble average of intracellular concentrations, is robust to the variability of individual bacteria and can be described by a simple deterministic model with a reasonable accuracy. Interestingly, the value of 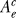 predicted by our model is of the same order of magnitude as the experimentally found values (150 and 900 nM) for the two QS systems of *Serratia liquefaciens* [36].

### Transition to QS in the Growing Population is Dynamic

**Figure 6:**
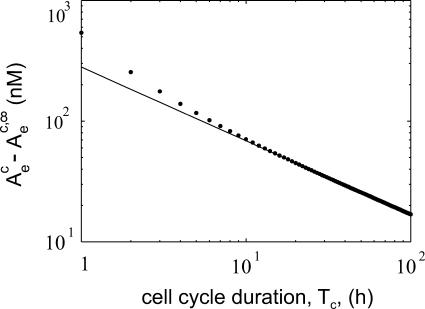
Critical Extracellular Concentration of Autoinducer Depends on the Growth Rate of a Bacterial Population The difference between the actual critical concentration *A_e_^c^* computed using the deterministic population model and the value predicted by the intracellular model *A_e_^c,∞^* is plotted against the duration of cell cycle *T_c_* (filled circles). As shown by the linear fit (solid line), *A_e_^c^* approaches *A_e_^c,∞^* according to the power law *A_e_^c^ − A_e_^c,∞^∝T^ − 0.61^_c_*.

The value of the critical AAI concentration 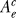 for the exponentially growing spatially homogeneous population is more than ten times larger than the one that follows from the case in Figure 4. This discrepancy arises because in the computation of both stochastic and deterministic values presented in Figure 4 it is implicitly assumed that a bacterium remains in the medium with a given extracellular AAI concentration for a practically infinite amount of time. In the growing population, *A_e_* rises exponentially together with the population density and the intracellular network does not have sufficient time to adjust to the extracellular environment. As a result, the transition to QS in a growing population always occurs in conditions far from stationary, and the values of the critical AAI concentration and the cell density threshold depend on the parameters of the population growth. The duration of the cell cycle that defines the population growth rate is one of the key parameters. The lower the population growth rate, the smaller the required 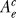 . In the unrealistic limit of an infinitely slowly growing population, the transition is guaranteed to occur at the value found for the static intracellular model (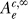 ≈ 48 nM). Figure 6 shows that the difference between the actual critical concentration 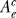 and its asymptotic value 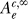 first decreases dramatically with the increase in the cell cycle duration *T_c_* and then slowly vanishes in accordance with an apparent power law 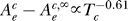 .

The threshold population density depends on 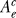 and a number of other population parameters, e.g., the spatial distribution of bacteria in the habitat (see below). The value of the cell density *N^c^* that has to be reached to achieve QS under specific environmental conditions can provide useful information on the possibility of achieving QS in an environmental niche. For example, experimental observation of the transition of an *Agrobacterium* population to QS in liquid medium can be problematic because of the large value of the predicted density threshold (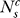 ≈ 2.0 × 10^9^ cells/ml by the stochastic model and 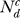 ≈ 2.82 × 10^9^ cells/ml by the deterministic approach). In response to depletion of the nutrients in the liquid medium, a typical *Agrobacterium* population begins the transition to the stationary growth phase at or even below the predicted *N^ c^* values [37]. The ensuing general stress response activates transcription of lactonase *attM,* which efficiently destroys autoinducer molecules and abrogates the transition to QS [37–39]. Thus, even in the nutrient-rich conditions of the optimized liquid medium it is not unlikely that the transition to the stationary phase precedes and, therefore, precludes the transition to QS.

### Spatial Heterogeneity Reduces Population Density Threshold

**Figure 7:**
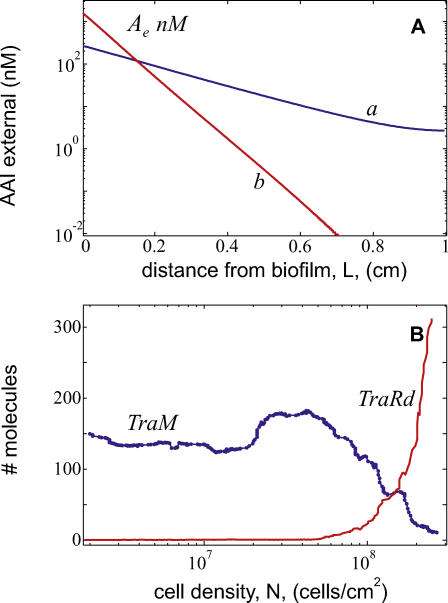
Transition to Quorum Sensing in a Model Bacterial Population Growing as an Attached Biofilm (A) Spatial gradient of autoinducer created by the biofilm after 7 h of growth at two different values of AAI diffusion coefficient: (a) 10^−5^ cm^2^/s and (b) 10^−6^ cm^2^/s. The *x-*axis is perpendicular to the plane of the biofilm, which is positioned at *x* = 0. (B) Intracellular copy numbers of TraRd and TraM averaged over 20 randomly selected bacteria versus the population density for the slower diffusion of AAI.

We interpreted the previous finding as an indication that the simplest experimental scenario of growth in a spatially homogeneous liquid medium is not ecologically relevant and the QS network is not “tuned on” to support the transition to QS in such conditions. Indeed, in nature, the transition to QS and subsequent bacterial conjugation take place in the thin but dense biofilm on the surface of a Ti-plasmid-induced plant tumor. In laboratory conditions, the experiments on detection and quantification of conjugation are normally performed in the quasi two-dimensional environment of polymer filters that provide bacteria with firm support for attachment and conjugation [40]. We therefore modified the stochastic population model to simulate an immotile bacterial population that grows exponentially on the surface of a filter that is placed at the bottom of a Petri dish and covered with 1 cm of an unstirred liquid medium. We also roughly estimated the transition to QS in the biofilm growing on the interface between the plant tumor and the soil using the same spatial layout but with the AAI diffusion coefficient reduced by a factor of ten. Although such simplistic approximation may not adequately represent complex conditions in the soil, it reveals a qualitative trend relevant for the goal of our study. In the absence of mechanical mixing and active bacterial motion, AAI excreted into the extracellular space freely diffuses into the medium and forms a sharp concentration gradient. As can be seen in the simulation results (Figure 7A), the AAI concentration drops exponentially from hundreds of nanomoles per liter in the plane of biofilm to barely detectable values on the medium boundary. Lower conductivity of the medium results in a steeper gradient, faster accumulation of AAI in the plane of biofilm, and reduced losses of AAI into the environment. Monitoring of the population-averaged intracellular variables shows that the transition to QS in the biofilm is not appreciably different from that in the bulk of the liquid medium (Figure 7B) and takes place at the similar critical extracellular AAI concentration 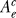 ≈ 615 nM. In contrast, the threshold population densities, 4.43 × 10^8^ cells/cm^2^ for the faster and 1.33 × 10^8^ cells/cm^2^ for the slower diffusion, are considerably lower than the homogeneous bulk value. From analysis of electron microscopy images of *Agrobacterium* biofilms [41], we calculated a typical cell density to be between 0.5 and 5 cells/μm^2^ (0.5–5.0 × 10^8^ cells/cm^2^). The values predicted by our model are thus well within the natural range and can be readily reached by a biofilm growing in the optimal nutrition conditions, e.g., on the feeding surface of a plant tumor.

### Conclusions

Using the phenomenon of bacterial QS as an enlightening example, we investigated the relationship between the dynamics of an intracellular molecular network and the population-wide phenotype that is controlled by this network. We first reconstructed the QS network of *Agrobacterium* Ti plasmids from experimental data and demonstrated that the network actually possesses the properties required for a gene expression switch, such as high sensitivity to environmental control and robustness to molecular noise. We then developed a stochastic model of a bacterial population to explore the transition to QS in a number of simple experimental scenarios, namely, during exponential growth in homogeneous liquid bulk and in an attached biofilm.

One of the long-standing questions in this field is whether all cells in a population produce autoinducer at the same rate and experience transition to QS simultaneously [2]. Our results predict that even in spatially homogeneous medium there is dramatic variability between the cells in the level of transcription factor and therefore the rate of production of autoinducer. This variability presumably arises from the fact that in a single cell the transition to QS, essentially an autocatalytic process, vastly amplifies the natural stochasticity of gene expression inherent in bacteria [42–44]. As a result, individual cells experience transition to QS in a wide range of extracellular concentrations of autoinducer and at varied levels of population density. Such nongenetic variability in the behavior of individual bacterial cells has been demonstrated to be advantageous for population survival in some cases, e.g., in the phenomenon of bacterial persistence under antibiotic treatment [45,46]. In our system, this variability may improve chances of Ti plasmid propagation within the biofilm when the density of donor cells falls short of the critical value. Although no direct evidence for this has been reported, such variability can explain why the appearance of conjugation between donors and recipients is observed emerging gradually over an extended cell density range [47]. Additional factors responsible for the widening of the transition range may result from spatial heterogeneity within the biofilm. In the present stochastic model we disregarded inhomogeneity of cell distribution in the plane of the biofilm. In future research it would be interesting to explore the influence of spatial heterogeneity typical of natural biofilms on the transition to QS.

Despite large variability between individual cells, a single population-wide value can be meaningfully defined for both critical concentration of autoinducer and the threshold population density for the transition to QS if the intracellular dynamics of individual cells is averaged over the population. These values are robust to stochasticity of individual bacteria and can be predicted with sufficient accuracy by a deterministic population model provided that spatial heterogeneity is insignificant. Our model demonstrates that when driven by exponential growth, the population transition to QS does not occur under steady state conditions and therefore the critical values depend on the parameters of population growth. Thus, the critical concentration of autoinducer depends on the growth rate.

Importantly, bacteria grown in different experimental conditions require different population densities to reach QS. Transition to QS in the bulk of the liquid medium appears to be the least favorable and requires much higher population density than transition in a biofilm. This result suggests potential ecological and evolutionary significance of the QS phenomenon for Ti plasmid propagation. In natural conditions a bacterial population dwells in a heterogeneous habitat with both bulk (e.g., soil) and the attracting surface (the plant–soil interface). Given the difference in cell density thresholds, it is likely that the transition to QS occurs in the surface-attached biofilm but not in the bulk. Therefore, it is tempting to speculate that the Ti plasmid utilizes QS to detect whether its bacterial host is firmly attached to the biofilm in close proximity to the source of nutrition (octopine) and, therefore, is in favorable conditions to initiate the conjugal transfer. Contribution of QS to the maturation of biofilms has been suggested for a number of species (for review see [2]). Interestingly, in our case QS does not influence any morphogenetic process in the biofilm but rather appears to detect the condition when the biofilm is sufficiently advanced in its formation. The requirement of biofilm formation for conjugation might be explained by the fact that solid support was found to be essential for the success of Ti plasmid transfer between the bacterial cells [47]. In addition, location inside a biofilm implies a high density of neighbors and therefore a high probability of finding a conjugation partner in close proximity.

Thus, our systems-level model provides experimentally testable quantitative predictions regarding both the dynamics of the intracellular control network and the population-wide characteristics of the transition to QS. Experimental verification of these predictions can be achieved, for example, by using a combination of classical genetic techniques and modern fluorescent confocal microscopy. One of the less obvious model predictions amenable to this approach is the existence of an appreciable intracellular pool of TraM in the off state. Insertion of a fluorescent reporter, like the green fluorescent protein or one of its derivatives, into some or all operons controlled by TraRd would result in the development of a sensitive gage to directly observe the dynamics of the transition to QS in vivo. With such a reporter it should be possible to observe bistability in the transition region by simultaneously monitoring populations of cells in the off and on states, e.g., as has recently been done in a study of the lactose utilization network [48]. Ability to monitor in vivo the copy number of TraRd or, complementary to it, concentration of TraM in a statistically significant number of bacterial cells would also allow one to measure the critical concentration of AAI and the threshold population density. The dependence of the autoinducer critical concentration on the growth rate is also a testable prediction. Our model predicts that if the duration of the cell cycle increases from 1 h to 2 h, 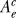 should drop almost by a factor of two.

In addition to producing these predictions, our study suggests an answer to the long-standing question regarding the ecological and evolutionary role of the QS phenomenon in the genetic conjugation of Ti plasmids. Finally, our analysis demonstrates how computational modeling connects multiple scales of biological phenomena, from the level of molecular networks to that of multicellular populations.

## Materials and Methods

### Mathematical formulation of intracellular dynamics.

We formulated the chemical kinetics of the *Agrobacterium* QS network as a system of 16 mass-action rate law equations with 30 chemical constants and the extracellular concentration of AAI as a free parameter. Complex processes of transcription and translation are represented in our formulation by cumulative reactive mechanisms:


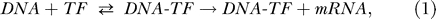



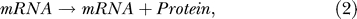


with three and one effective reaction constants, respectively. The action of the putative AAI importer is modeled according to the standard enzymatic mechanism:


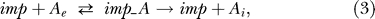


where *A_e_* and *A_i_* are the extracellular and intracellular concentrations of AAI, respectively. Detailed formulation of each model equation is given in Table 1.

**Table 1:**
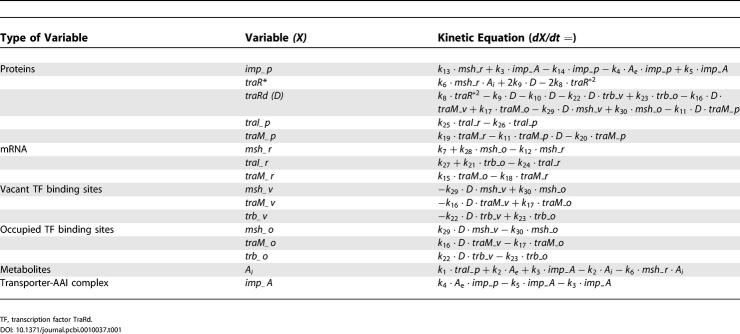
Model Variables and Equations

### Kinetic constants.

**Figure 8:**
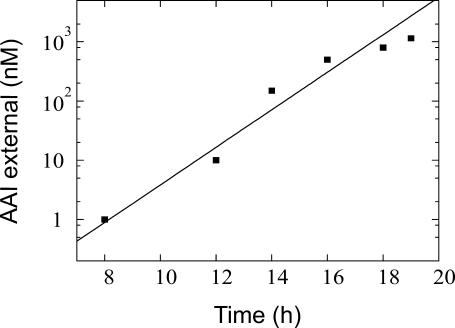
Extracellular Concentration of AAI in the Culture of the TraM-Defective Mutant Strain K588 versus Time in the Exponential Growth Phase The model (solid line) is fitted to the experimental data (filled squares). All parameters are as in Table S1, except *k*_11_, which is set to zero to model the inability of mutated TraM to sequester TraRd.

We extracted a number of crucial model parameters, e.g., lifetime of TraRd and reaction constant of TraRd and TraM, directly from the literature. Many parameters of a more general nature, such as velocities of transcription and translation, are not reported for our system and were estimated based on values obtained for other prokaryotic systems. We estimated the average volume of a bacterial cell *V_b_* to be 1.4 × 10^−13^ cm^3^ (a cylinder with diameter 0.3 μm and length 2 μm) using high-quality electronic microscopy images of *Agrobacterium* colonies [41]. Based on this value we converted all concentrations from moles per liter (M) to molecules per cell (one molecule per cell corresponds to approximately 12 nM) and adjusted kinetic constants appropriately. A full set of the constants used in this work is presented in Table S1. To ensure validity of the population model predictions, it is essential to correctly evaluate the level of AAI production by a bacterial cell. We achieved this by fitting our model to the experimental data obtained for the conjugation constitutive mutant strain K588 [37] (Figure 8). This mutant produces copious amounts of AAI that can be readily detected in the medium throughout population growth (see Results and Discussion).

### Sensitivity analysis.

We performed a local analysis of sensitivity of the stationary states of the full deterministic model to variation of the model parameters using the formalism of the metabolic control analysis [34] that recently has been extended for the analysis of signal transduction and gene expression networks [49,50]. In this framework, the sensitivity of the stationary concentrations of intracellular components *C_i_* to variation of the model parameters *k_j_* is given by the concentration response coefficients:


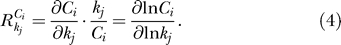


These coefficients were computed using Jarnac, which was integrated into Systems Biology Workbench, a freely available software platform [51].

### Reduction of the full model.

We used standard methods of chemical kinetics to reduce the dimensionality of the full QS network model to only two equations describing the dynamics of TraRd and intracellular AAI. We first eliminated variables describing vacant and occupied TraRd binding sites (see Table 1), assuming that the quasistationary approximation holds for these variables. This assumption results in the effective Michaelis–Menten approximation for all transcription events. We simultaneously introduced corresponding Michaelis–Menten constants, which in this case are the coefficients of dissociation for TraRd binding to respective *cis*-regulatory elements, e.g., 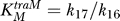 . Thus, for example, the equation for *traM* mRNA becomes:


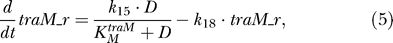


where *D* represents concentration of TraRd as in Table 1. In a similar fashion we eliminated the variable *imp_A* and introduced another Michaelis–Menten constant, 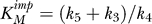 . We then applied quasistationary approximation to all three mRNA species as well as to the proteins Imp, TraI, and TraM and the monomeric complex TraR*. This allowed us to express all of the remaining variables through the concentrations of TraRd and AAI. For example, from the previous equation the quasistationary concentration of *traM* mRNA is:


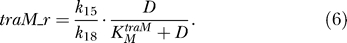


Finally, we obtained the reduced model with two equations:


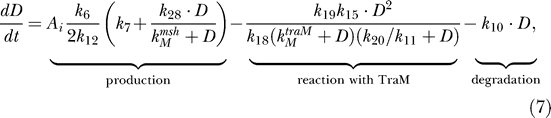



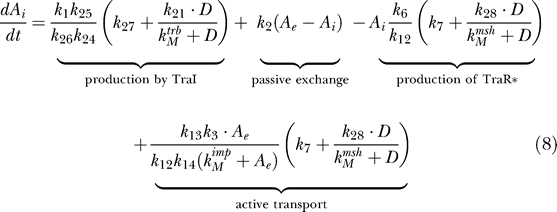


### Population model.

Since the full model for the intracellular dynamics is expressed entirely in mass-action rate law equations, it can be simulated with both deterministic and stochastic methods. To model the transition to QS in the exponentially growing bacterial population deterministically, we complemented the intracellular model with two equations describing the dynamics of cell density *N* and extracellular autoinducer *A_e_*:


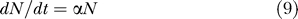



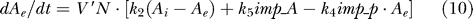


where *V′* is the ratio of the bacterial cell volume *V_b_* to the full volume occupied by the population suspended in the medium *V_e_* (1 ml) and the expression in square brackets represents the exchange of AAI between a bacterium and the environment (see Table 1 for details).

In the stochastic formulation, each bacterium is represented by a separate agent endowed with an independent stochastic realization of the QS network model. We assumed that bacteria can either randomly move in the medium (planktonic form) with effective diffusion coefficient *D_b_* = 10^−6^ cm^2^/s [52] or remain immotile (attached form). Both forms exchange AAI with the surrounding medium according to the model equations. In the extracellular environment AAI is represented by a spatially dependent continuous concentration field and its spatiotemporal evolution is modeled by the diffusion equation. While the diffusion coefficient of AAI is not known, from the size of the molecule we estimated that AAI diffuses in the liquid bacterial culture with *D_A_* = 10^−5^ cm^2^/s. Cells periodically divide, resulting in two replicas that are exactly identical at the moment of division and diverge thereafter. In both deterministic and stochastic representations average cell cycle is 1 h (α = 1.9 × 10^−4^ s^−1^) according to the estimate based on our experimental data.

### Computational realization.

All deterministic simulations were performed with Matlab (MathWorks, Natick, Massachusetts, United States). The stochastic model of the intracellular network was simulated with the exact Gillespie algorithm [53] using public-domain cell-modeling software Cellware [54,55]. To model stochastic population dynamics we developed a dedicated parallel software platform [56] that manages dynamically growing bacterial populations and utilizes Cellware to compute the intracellular dynamics for each cell agent. The platform also simulates cell motion, exchange of AAI with the medium, and diffusion of AAI in the medium. The software was implemented in C++ and MPI to run on a commodity Linux cluster. All codes used in this work are freely available on request.

## Supporting Information

Table S1 Model Parameters (121 KB DOC)

### Accession Numbers

The NCBI Entrez Protein (http://www.ncbi.nlm.nih.gov/entrez/query.fcgi?db=Protein) accession numbers for the proteins discussed in this paper are TraI (AAB95104), TraM (AAC28120), and TraR (AAC28121). The NCBI Entrez Gene (http://www.ncbi.nlm.nih.gov/entrez/query.fcgi?db=gene) accession numbers for the genes discussed in this paper are *ophABCDE* (1224221–1224225), *traI* (1224280), *traM* (1224219), and *traR* (1224220).

## Abbreviations

AAI: *Agrobacterium* autoinducer
Imp: *Agrobacterium* autoinducer importer
QS: quorum sensing
TraRd: TraR–*Agrobacterium* autoinducer complex dimer
TraR*: TraR–*Agrobacterium* autoinducer complex
