# Supplementary material for: Transition to Quorum Sensing in an Agrobacterium Population: A Stochastic Model
Source: PLoS Comput Biol. 2005 Sep 16;1(4):e37. doi: 10.1371/journal.pcbi.0010037 (PMC1214540; doi:10.1371/journal.pcbi.0010037)
Supplement: Supplementary file 1 [file pcbi.0010037.st001.doc]

**Supplementary Table 1. Model parameters**

| Kinetic Constant | Legend | Value/Range | References | Used in simulation^1,2^ | Sensitivity^3^ |
| --- | --- | --- | --- | --- | --- |
| *k_1_* | production of AAI by TraI | 0.02-0.4 s^-1^ | [57,58] | 0.3 s^-1^ | 1.05  1.72  0.47 |
| *k_2_* | passive diffusion of AAI through the cell wall | 0.1-0.4 s^-1^ | [59,60] | 0.14 s^-1^ | -1.05  -4.87  -1.88 |
| *k_3_* | active transport of AAI: maximal velocity | 0.02-0.6 s^-1^ | [61–63] | 0.4 s^-1^ | 0.00  0.02  0.01 |
| *k_4_* | active transport of AAI: binding to Imp, "on" rate | 1.0-8.0 10^6^ M^-1^s^-1^ | [61–63] | 0.04 m^-1^s^-1^ | 0.00  3.14  1.41 |
| *k_5_* | active transport of AAI: binding to Imp, "off" rate | 0.5-5.0 10^-3^ s^-1^ | [61–63] | 2.0 10^-3^ s^-1^ | 0.00  -0.02  -0.01 |
| *k_6_* | formation of TraR* complex between TraR and AAI | 0.1-5.0 10^3^ M^-1^ s^-1^ | [64,65] | 3.56 10^-5^  m^-1^s^-1^ | 1.05  4.88  1.88 |
| *k_7_* | transcription rate of the occ operon: maximal velocity | 1.0-4.0 10^-10^ M s^-1^ | [65–67] | 1.5 10^-2^  m s^-1^ | 0.99  5.33  1.34 |
| *k_8_* | dimerization of TraR* | 0.5-5.0 10^5^ M^-1^s^-1^ | [18–20,68] | 1.0 10^-3^ m^-1^s^-1^ | 0.00  0.00  0.00 |
| *k_9_* | dissociation of TraRd | 0.5-5.0 10^-4^ s^-1^ | [18–20,68] | 1.0 10^-4^ s^-1^ | 0.00  0.00  0.00 |
| *k_10_* | degradation of TraRd | 1.6-3.3 10^-4^ s^-1^ | [18,20] | 3.3 10^-4^ s^-1^ | 0.00  -0.01  -1.00 |
| *k_11_* | formation of complex between TraRd and TraM | 7 10^4^ M^-1^s^-1^ | [24] | 8.3 10^-4^ m^-1^s^-1^ | -0.78  -0.89  0.00 |
| *k_12_* | degradation of msh mRNA | 1.4 -7.0 10^-3^ s^-1^ | [69] | 6.0 10^-3^ s^-1^ | -1.05  -8.01  -3.29 |
| *k_13_* | translation of msh mRNA | 0.8-3.2 10^-2^ s^-1^ | [65–67] | 1.6 10^-2^ s^-1^ | 0.00  3.14  1.41 |
| *k_14_* | degradation of Imp | 0.2-4.0 10^-4^ s^-1^ | [64] | 10^-4^ s^-1^ | 0.00  -3.14  -1.41 |
| *k_15_* | transcription of traM: maximal velocity | 1.0-4.0 10^-10^ M s^-1^ | [65,66,70] | 1.6 10^-2^  m s^-1^ | -1.04  -4.87  -0.88 |
| *k_16_* | transcription of traM: binding of TraRd, "on" rate | 0.8-2.5 10^6^ M^-1^s^-1^ | [18,71,72] | 1.4 10^-2^ m^-1^s^-1^ | -0.99  -2.95  0.00 |
| *k_17_* | transcription of traM: binding of TraRd, "off" rate | 0.5-5.0 10^-2^ s^-1^ | [18,71,72] | 9.26 10^-3^ s^-1^ | 0.99  2.95  0.00 |
| *k_18_* | degradation of traM mRNA | 1.4 -7.0 10^-3^ s^-1^ | [69] | 1.6 10^-3^ s^-1^ | 1.04  4.87  0.88 |
| *k_19_* | translation of traM mRNA | 0.8-3.2 10^-2^ s^-1^ | [65–67] | 1.92 10^-2^ s^-1^ | -1.04  -4.87  -0.88 |
| *k_20_* | TraM degradation | 0.2-4.0 10^-4^ s^-1^ | [64] | 8.0 10^-5^ s^-1^ | 0.78  0.89  0.00 |
| *k_21_* | transcription of trb operon: maximal velocity | 1.0-4.0 10^-10^ M s^-1^ | [65,66,70] | 1.6 10^-2^  m s^-1^ | 0.76  1.65  0.46 |
| *k_22_* | transcription of trb operon: binding of TraRd, "on" rate | 0.8-2.5 10^6^ M^-1^s^-1^ | [18,71,72] | 2.68 10^-2^ m^-1^s^-1^ | 0.73  1.08  0.00 |
| *k_23_* | transcription of trb operon: binding of TraRd, "off" rate | 0.5-5.0 10^-2^ s^-1^ | [18,71,72] | 2.2 10^-2^ s^-1^ | -0.73  -1.08  0.00 |
| *k_24_* | degradation of traI mRNA | 1.4 -7.0 10^-3^ s^-1^ | [69] | 6.0 10^-3^ s^-1^ | -1.05  -1.72  -0.47 |
| *k_25_* | translation of traI mRNA | 0.8-3.2 10^-2^ s^-1^ | [65–67] | 1.6 10^-2^ s^-1^ | 1.05  1.72  0.47 |
| *k_26_* | TraI degradation | 0.2-4.0 10^-4^ s^-1^ | [64] | 10^-4^ s^-1^ | -1.05  -1.72  -0.47 |
| *k_27_* | traI constitutive expression | 0.9-4.5 10^-12^ M s^-1^ | set to yield 1-5 molecules traI/cell in the absence of octopine | 2.3 10^-4^ m s^-1^ | 0.29  0.07  0.01 |
| *k_28_* | transcription of msh operon: maximal velocity | 1.0-4.0 10^-10^ M s^-1^ | [65,66,70] | 2.2 10^-2^  m s^-1^ | 0.05  2.68  1.96 |
| *k_29_* | transcription of msh operon: binding of TraRd, "on" rate | 0.8-2.5 10^6^ M^-1^s^-1^ | [18,71,72] | 2.9 10^-3^ m^-1^s^-1^ | 0.05  1.76  0.00 |
| *k_30_* | transcription of msh operon: binding of TraRd, "off" rate | 0.5-5.0 10^-2^ s^-1^ | [18,71,72] | 2.4 10^-3^ s^-1^ | -0.05  -1.76  0.00 |

^1^ Unless directly stated otherwise, this value is used in all simulations presented throughout this paper.

^2^ Values are adjusted for concentrations expressed as molecules per cell (abbreviated as m) using conversion factor $8.43\cdot10^{7}m/M$.

^3^ Sensitivity coefficients are computed as described in Materials and Methods at the three values of extracellular concentration of AAI: 0, 42 and 120 *nM* respectively.

Additional References

57. Parsek MR, Val DL, Hanzelka BL, Cronan JE Jr, Greenberg EP (1999) Acyl homoserine-lactone quorum-sensing signal generation. Proc Natl Acad Sci U S A 96: 4360–4365.

58. Schaefer AL, Val DL, Hanzelka BL, Cronan JE Jr, Greenberg EP (1996) Generation of cell-to-cell signals in quorum sensing: Acyl homoserine lactone synthase activity of a purified *Vibrio fischeri* LuxI protein. Proc Natl Acad Sci U S A 93: 9505–9509.

59. Kaplan HB, Greenberg EP (1985) Diffusion of autoinducer is involved in regulation of the *Vibrio fischeri* luminescence system. J Bacteriol 163: 1210–1214.

60. Rubinow SI (2002) Introduction to mathematical biology. New York: Dover Publications. 386 p.

61. Detmers FJ, Kunji ER, Lanfermeijer FC, Poolman B, Konings WN (1998) Kinetics and specificity of peptide uptake by the oligopeptide transport system of *Lactococcus lactis*. Biochemistry 37: 16671–16679.

62. Fang G, Konings WN, Poolman B (2000) Kinetics and substrate specificity of membrane-reconstituted peptide transporter DtpT of *Lactococcus lactis*. J Bacteriol 182: 2530–2535.

63. Picon A, Kunji ER, Lanfermeijer FC, Konings WN, Poolman B (2000) Specificity mutants of the binding protein of the oligopeptide transport system of *Lactococcus lactis*. J Bacteriol 182: 1600–1608.

64. Pratt JM, Petty J, Riba-Garcia I, Robertson DH, Gaskell SJ, et al. (2002) Dynamics of protein turnover, a missing dimension in proteomics. Mol Cell Proteomics 1: 579–591.

65. Iyer V, Struhl K (1996) Absolute mRNA levels and transcriptional initiation rates in *Saccharomyces cerevisiae.* Proc Natl Acad Sci U S A 93: 5208–5212.

66. Kierzek AM, Zaim J, Zielenkiewicz P (2001) The effect of transcription and translation initiation frequencies on the stochastic fluctuations in prokaryotic gene expression. J Biol Chem 276: 8165–8172.

67. Watson JD, Hopkins NH, Roberts JW, Steitz JA, Weiner AM (2003) Molecular biology of the gene, 5th ed. New York: Benjamin Cummings. 732 p.

68. Luo ZQ, Smyth AJ, Gao P, Qin Y, Farrand SK (2003) Mutational analysis of TraR. Correlating function with molecular structure of a quorum-sensing transcriptional activator. J Biol Chem 278: 13173–13182.

69. Hambraeus G, von Wachenfeldt C, Hederstedt L (2003) Genome-wide survey of mRNA half-lives in *Bacillus subtilis* identifies extremely stable mRNAs. Mol Genet Genomics 269: 706–714.

70. Dundr M, Hoffmann-Rohrer U, Hu Q, Grummt I, Rothblum LI, et al. (2002) A kinetic framework for a mammalian RNA polymerase in vivo. Science 298: 1623–1626.

71. Kugel JF, Goodrich JA (2000) A kinetic model for the early steps of RNA synthesis by human RNA polymerase II. J Biol Chem 275: 40483–40491.

72. Narayan S, Widen SG, Beard WA, Wilson SH (1994) RNA polymerase II transcription. Rate of promoter clearance is enhanced by a purified activating transcription factor/cAMP response element-binding protein. J Biol Chem 269: 12755–12763.
